# Use of natural biotechnological processes to modify the nutritional properties of bean-based and lentil-based beverages

**DOI:** 10.1038/s41598-023-44239-8

**Published:** 2023-10-09

**Authors:** Patrycja Cichońska, Joanna Bryś, Małgorzata Ziarno

**Affiliations:** 1 Department of Food Technology and Assessment, Institute of Food Science, Warsaw University of Life Sciences - SGGW (WULS-SGGW), Nowoursynowska 159C St., 020776 Warsaw, Poland; 2 Department of Chemistry, Institute of Food Science, Warsaw University of Life Sciences - SGGW (WULS-SGGW), Nowoursynowska 159C St., 020776 Warsaw, Poland

**Keywords:** Bacteria, Plant biotechnology

## Abstract

The market for plant-based beverages (PBBs) is relatively new; hence, to enable its further development, it is important to use new raw materials and improve production technology. The use of natural biotechnological processes can diversify the segment of PBBs, which may offer products with better functionality than those available in the market. Therefore, the present study aimed to determine the effects of fermentation and germination on the nutritional properties of bean-based beverages (BBs) and lentil-based beverages (LBs). The applied processes significantly (p ≤ 0.05) influenced the characteristics of PBBs. Fermentation improved the antioxidant properties (e.g., by increasing the level of 1,1-diphenyl-2-picrylhydrazyl radical scavenging activity by 2–6% and 3–7% for BBs and LBs, respectively) and modified the fatty acid (FA) profile of PBBs. This process increased the share of polyunsaturated FAs in the sn2 position in triacylglycerols, which may promote its absorption in the intestine. The simultaneous use of germination and fermentation was most effective in decreasing oligosaccharide content (< 1.55 mg/kg), which may reduce digestive discomfort after consuming PBBs. We recommend that the designing of innovative legume-based beverages should include the application of fermentation and germination to obtain products with probiotic bacteria and improved nutritional properties.

## Introduction

Natural biotechnological processes have been used for centuries to increase the nutritional value of foods and extend their shelf life. It is hypothesized that as farming started to replace hunting and gathering around 10,000 years ago, humans began to produce fermented foods and beverages^1^. Sprouting (also known as germination) of seeds has been known for a very long time, mainly in Eastern countries where germinated plants have been used in traditional cuisine^2^. Presently, these techniques are being successfully used to process plant and animal products^3,4^; thus, it may be beneficial to use these techniques to produce novel healthy foods.

Plant-based diets are becoming increasingly popular in the global food market; consequently, plant-based substitutes for animal products are in great demand^5^. The most popular products in this food group are milk substitutes, which are also known as plant-based beverages (PBBs). PBBs are water-soluble extracts of shredded plant materials (e.g., legumes, seeds, cereals, pseudocereals, and nuts) in the form of colloidal suspensions or emulsions^6,7^. Among the nondairy matrices, legumes are the least explored ones, with the majority of studies focused on soybean alone^5,8^. However, the market share of soy beverages is decreasing because of health concerns related to genetically modified organisms, allergens, high levels of isoflavones, and CO_2_ footprint^9^. Thus, there is an opportunity to expand and diversify this segment by using other legumes that may provide better functionality and nutrition than soy. Hence, it is necessary to gain more knowledge regarding the processing, functionality, and health benefits and risks of beverages prepared using other legumes^10^.

Currently, legume consumption is minimal because of limitations such as taste, availability, flatulence factor, and lack of knowledge regarding the preparation process. Designing new legume-based beverages is a beneficial approach that can overcome these limitations by offering modern convenience food^10^. Various methods have been used to improve the properties of legumes, such as soaking, cooking, fermentation, and germination^11^. The use of these processes, particularly in combination, can provide improved nutritional value, aroma, taste, texture, stability, and safety against microbial contamination^7,12^.

The market for PBBs is relatively new; therefore, to enable its further growth and development, it is essential to improve the technology of producing PBBs. A goal of the food industry is to develop new, ecofriendly processing strategies to reduce energy consumption and maximize the value of raw materials^13^. The answer to this may be the use of natural biotechnological processes such as germination and fermentation, which can increase the attractiveness of legume-based beverages and does not require the use of technologies with limited industrial application due to high energy consumption and cost related to complex equipment purchase^13,14^.

Previous research on the properties of legume-based beverages other than those made from soybean does not provide clear recommendations on using processes to improve product properties and overcoming barriers to their consumption. Moreover, to date, the effects of a combination of different biotechnological processes on the properties of bean-based beverages (BBs) and lentil-based beverages (LBs) have not been studied. The study focused on a BBs and LBs due to their potential to produce highly nutritious PBBs from legumes other than soy. This is the result of the high content of protein, carbohydrates, dietary fiber, and bioactive ingredients (such as polyphenols) in beans and lentils^10,11^. We believe that these legumes hold promise in addressing the growing demand for sustainable and nutritious food alternatives, due to their nutritional richness, diversity, cultural significance, availability, and the potential for further advancement in the development of protein-enriched plant-based foods. Therefore, the present study aimed to assess the possibility of using natural biotechnological processes, i.e., fermentation and germination, to improve the nutritional properties of BBs and LBs. The influence of the studied processes on fermentation (pH and population of lactobacilli and bifidobacteria), nutritional profile (carbohydrate content, total share of fatty acids, fatty acid profile, and positional distribution of fatty acids in triacylglycerols (TAGs)), and antioxidant properties (total phenolic content and 1,1-diphenyl-2-picrylhydrazyl (DPPH) radical scavenging activity (RSA)) of BB and LB was analyzed. The changes in the tested properties of the PBBs after their production and after 21 days of refrigerated storage at 6 °C were analyzed. This study also aimed to offer recommendations for the use of fermentation and germination for producing innovative legume-based beverages.

## Materials and methods

### Materials and experimental design

The PBBs used in our study were prepared from white kidney beans “Piękny Jaś Karłowy” (Lestello Sp. z o.o., Cmolas, Poland) and brown lentils (Natural Expert, Bialystok, Poland). The nutritional values of the tested beans and lentils are shown in Supplementary Table S1 online, according to the product label.

Three industrial freeze-dried starter cultures were used: Beaugel Soja 1 (Ets Coquard, Villefranche-sur-Saône, France), which comprised *Lactobacillus casei* (currently classified as *Lacticaseibacillus casei*), *Streptococcus thermophilus*, and *Lactobacillus delbrueckii* subsp. *bulgaricus*; YO-MIX 207 LYO 500 DCU (DuPont™ Danisco, Copenhagen, Denmark), which comprised *S. thermophilus*, *L. delbrueckii* subsp. *bulgaricus*, *Lactobacillus acidophilus*, and *Bifidobacterium lactis*; and ABY-3 (Chr. Hansen, Hørsholm, Denmark), which comprised *L. acidophilus* La-5, *Bifidobacterium animalis* subsp. *lactis* BB-12, *S. thermophilus*, and *L. delbrueckii* subsp. *bulgaricus*. These starter cultures were selected to determine the differences between fermentation with a starter culture containing basic yoghurt bacteria (Beaugel Soja 1) and fermentation with starter cultures enriched with different strains of bifidobacteria (YO-MIX 207 and ABY-3).

Two variants of each PBB were prepared: from germinated (G) and non-G beans and lentils. The experimental design used in the study was based on three factors: germination, starter culture used for fermentation, and storage period at 6 °C (Table 1). The experiment was conducted in duplicate. Supplementary Table S2 online shows the physicochemical characteristics of basic PBBs derived from G beans (BBG), non-G beans (BB), G lentils (LBG), and non-G lentils (LB).

**Table 1:** The explanation of the sample codes for tested PBBs.

Sample code	Germination	Fermentation	Storage at 6 °C
BB0/LB0	−	−	−
BB100/LB100	−	Beaugel Soja 1	−
BB101/LB101	−	YO-MIX 207	−
BB102/LB102	−	ABY-3	−
BB0s/LB0s	−	−	21 days
BB100s/LB100s	−	Beaugel Soja 1	21 days
BB101s/LB101s	−	YO-MIX 207	21 days
BB102s/LB102s	−	ABY-3	21 days
BBG0/LBG0	+	−	−
BBG100/LBG100	+	Beaugel Soja 1	−
BBG101/LBG101	+	YO-MIX 207	−
BBG102/LBG102	+	ABY-3	−
BBG0s/LBG0s	+	−	21 days
BBG100s/LBG100s	+	Beaugel Soja 1	21 days
BBG101s/LBG101s	+	YO-MIX 207	21 days
BBG102s/LBG102s	+	ABY-3	21 days

### Preparation of PBBs

Germination was performed in a sprouter at 25 °C for 72 h (water was changed every 24 h). G and non-G beans and lentils were sterilized at 121 °C for 15 min, mixed with drinking water at the ratio of 1:9 (m/m), and blended until a homogeneous mass was obtained. The resulting mass was filtered through a sieve with 0.1 mm mesh size and then homogenized using the laboratory mixer L4R (Silverson, Chesham, UK). The prepared PBBs were then sterilized at 121 °C for 15 min.

### Fermentation of PBBs

Inoculums were prepared by dissolving the freeze-dried starter cultures in a sterile saline solution. The PBBs were inoculated with 1.0% (m/m) of starter cultures (final cell density of around 6–7 log CFU/mL) and incubated at 45 °C for 6 h. After fermentation, the PBBs were refrigerated at 6 °C and stored for 21 days. The non-stored PBBs were frozen at − 18 °C until analysis.

### Active acidity and microflora population analysis

The active acidity was determined by measuring the pH of the PBBs using a calibrated digital CPO-505 pH meter (Elmetron, Zabrze, Poland). The measurement was conducted twice.

Microflora population was analyzed by the culture method in Petri dishes. De Man, Rogosa, and Sharpe (MRS) agar (Merck, Darmstadt, Germany) was used to determine the viable population of lactobacilli. M17 agar (Merck) was used to determine the viable population of *S. thermophilus*. Bifidus Selective Medium (BSM) agar (Merck) was used to determine the viable population of bifidobacteria. The measurement was conducted twice. Petri dishes were incubated at 37 °C for 72 h under aerobic conditions for M17 medium and under anaerobic conditions for MRS and BSM media. After incubation, the number of colonies was counted, and CFU/mL was calculated. The result was expressed as a logarithm of the total cell count.

### Carbohydrate content of PBBs

The carbohydrate content was determined by high-performance liquid chromatography (HPLC) coupled with a refractive index detector (RID). The sample was prepared according to the method of Ziarno et al.^15^ without any modifications. The analytes were separated using an HPLC kit equipped with DeltaChrom™ pumps, an S 6020 needle injection valve dosing loop (Sykam, Fürstenfeldbruck, Germany), a DeltaChrom™ temperature control unit column temperature controller (Sykam), and a 05397–51 Cosmosil Sugar-D column (250 × 4.6 mm, 5 µm; Cosmosil, Nacalai Tesque, Kyoto, Japan) secured by a pre-column 05394–81 Cosmosil Guard Column Sugar-D (10 × 4.6 mm, 5 µm, Cosmosil). The analytes were detected with an S3580 RID (Sykam). The chromatographic analysis parameters were the same as those described by Ziarno et al.^15^ without any modifications. The analysis was performed in duplicate. After the analysis, the carbohydrates were identified by comparison with the retention times of selected carbohydrate standards, including glucose, sucrose, raffinose, stachyose, and verbascose (Sigma-Aldrich, Burlington, VT, USA).

### Analysis of antioxidant properties

The antioxidant properties of the PBBs were analyzed by determining total phenolic content (TPC) and the level of DPPH RSA according to the method of Zhao and Shah^16^ with some modifications. The PBB extracts were prepared by mixing 2 mL of the sample with 4 mL of 80% methanol; the extract was then vortexed for 1 min and centrifuged in an MPW-350R centrifuge (MPW Med. Instruments) at 4000 × *g* for 20 min at 4 °C. The precipitate was then extracted twice with 2 mL of 80% methanol, and the supernatants were pooled. All supernatants were filtered through a syringe filter (Merck) with 0.45 µm pore size and were used as the antioxidant extracts (AEs) to estimate TPC and DPPH RSA.

TPC (analyzed by the Folin–Ciocalteu method) and the efficiency of the PBBs to scavenge DPPH radicals were determined according to the method of Zhao and Shah^16^ without any modifications. The analyses were performed in duplicate. TPC was estimated by comparing the absorbance of the AEs with a calibration curve constructed using a gallic acid standard (0.5–2.5 mg/mL) and was expressed as milligrams of gallic acid (GAE)/mL of PBBs. RSA was expressed as percent DPPH inhibition using the following Eq. (1):

1 $$ {\text{DPPH RSA }}\left( \% \right) \, = \, \left[ {\left( {{\text{A}}_{0} {-}{\text{ A}}_{{\text{s}}} } \right)/{\text{A}}_{0} } \right] \, \times { 1}00, $$

where A_0_ is the absorbance of a methanolic solution of DPPH radicals without sample extract and A_s_ is the absorbance of PBBs.

### Analysis of fatty acid (FAs) profile

The PBBs were first extracted using the Folch method^17^. The FAs profile was analyzed according to the method of Ziarno et al.^18^ by using a gas chromatography system (YL6100 GC) with an installed flame ionization detector (Young Lin Instrument Co., Ltd., Anyang, Korea) and a MEGA-10 capillary column (ID 0.25 mm, film thickness 0.25 μm, length 60 m, MEGA S.r.l., Legnano, Italy). FAs were detected based on the retention time by comparison with a selected external standard (Fatty Acid Methyl Esters Standard Mixture, Merck). The analysis was performed in duplicate. The share of the analyzed FA in the FA profile was estimated by determining the area of the peaks in the chromatogram. The analysis was performed for basic PBBs obtained from G and non-G beans and lentils and for beverages fermented with the ABY-3 starter culture, which showed the highest acidification activity.

### Analysis of the positional distribution of FAs in TAGs

The hydrolysis of TAGs and the analysis of the positional distribution of FAs in TAGs were conducted and calculated according to the method of Ziarno et al.^18^ without any modifications. The analysis was performed for basic PBBs obtained from G and non-G beans and lentils and for beverages fermented with the ABY-3 starter culture (BB102/LB102, BB102s/LB102s, BBG102/LBG102, and BBG102s/LBG102s), which showed the highest acidification activity.

### Statistical analysis

Statistica version 13.1 software (StatSoft, Krakow, Poland) was used to analyze the data obtained in the experiments. Analysis of variance (ANOVA) was performed to determine the effect of germination (GM), fermentation (C), and storage (S) on the obtained results. Differences were considered significant at α = 0.05 based on Tukey’s test.

## Results and discussion

### Active acidity and viable bacterial population

Legume-based beverages provide a favorable matrix for fermentation; however, the process typically requires a longer time (approximately 12–48 h) for completion as compared to milk fermentation (approximately 4–6 h)^6,19^. During the fermentation of food (including milk and milk substitutes), it is beneficial to maintain pH in the range of 4.3–4.7; an acidic pH can protect the product from microbial contamination and retain favorable organoleptic properties related to the concentration of organic acids and other flavor-forming components^20,21^.

Table 2 shows the pH values and the population of lactic acid bacteria (LAB) and bifidobacteria in the tested PBBs. All fermented samples of PBBs showed a significant decrease in pH, which indicated the metabolic activity of microorganisms in all tested starter cultures. In all tested PBBs, no significant changes in pH were observed after storage. Fermentation was the primary factor that affected the obtained values (η^2^ ≈ 0.925–0.989).

**Table 2:** The pH and population of lactic acid bacteria and bifidobacteria in the tested PBBs. Table shows mean values and standard deviations (SD) range, and statistics ANOVA (η^2^—coefficient indicating the extent of the effect of factors G, C and S).

Sample code^1^	pH	Bacteria population [log10 CFU/mL]		
		Lactobacilli	*S. thermophilus*	Bifidobacteria
Bean-based beverages				
BB0	6.03 ± 0.32^b^	nd	nd	nd
BB100	4.79 ± 0.17^a^	5.99 ± 0.05^b^	9.60 ± 0.07^d^	nd
BB101	4.59 ± 0.36^a^	7.17 ± 0.06^cd^	8.16 ± 0.13^cd^	8.07 ± 0.22^b^
BB102	4.39 ± 0.25^a^	7.50 ± 0.02^de^	9.53 ± 0.18^d^	7.86 ± 0.08^ab^
BB0s	5.96 ± 0.38^b^	nd	nd	nd
BB100s	4.73 ± 0.25^a^	5.25 ± 0.21^a^	6.87 ± 0.11^b^	nd
BB101s	4.39 ± 0.55^a^	7.28 ± 0.09^cd^	8.18 ± 0.23^cd^	7.52 ± 0.13^a^
BB102s	4.38 ± 0.26^a^	7.49 ± 0.04^de^	7.72 ± 0.09^c^	7.43 ± 0.24^a^
BBG0	6.35 ± 0.06^b^	nd	nd	nd
BBG100	4.74 ± 0.24^a^	5.86 ± 0.11^b^	7.72 ± 0.18^c^	nd
BBG101	4.30 ± 0.07^a^	7.17 ± 0.18^cd^	8.08 ± 0.20^cd^	7.76 ± 0.13^ab^
BBG102	4.27 ± 0.22^a^	7.73 ± 0.11^de^	7.72 ± 0.17^c^	7.72 ± 0.18^ab^
BBG0s	6.30 ± 0.02^b^	nd	nd	nd
BBG100s	4.72 ± 0.06^a^	5.15 ± 0.07^a^	6.14 ± 0.19^a^	nd
BBG101s	4.40 ± 0.18^a^	7.47 ± 0.09^de^	8.32 ± 0.18^d^	7.74 ± 0.14^ab^
BBG102s	4.31 ± 0.13^a^	7.03 ± 0.08^c^	7.71 ± 0.11^c^	7.42 ± 0.13^a^
Statistics ANOVA. η^2^ [ −]				
GM	ns	ns	ns	ns
C	0.925	0.995	0.969	0.999
S	ns	0.211	0.265	0.261
Lentil-based beverages				
LB0	6.36 ± 0.08^e^	nd	nd	nd
LB100	4.36 ± 0.04^cd^	5.73 ± 0.10^ab^	8.60 ± 0.08^e^	nd
LB101	4.43 ± 0.02^abcd^	6.15 ± 0.17^bc^	7.65 ± 0.19^cd^	6.89 ± 0.06^a^
LB102	4.38 ± 0.01^abcd^	6.34 ± 0.10^cd^	8.62 ± 0.11^e^	6.75 ± 0.14^a^
LB0s	6.27 ± 0.03^e^	nd	nd	nd
LB100s	4.67 ± 0.08^d^	5.87 ± 0.18^abc^	7.28 ± 0.18^c^	nd
LB101s	4.46 ± 0.08^abcd^	7.23 ± 0.15^ef^	7.69 ± 0.13^ cd^	8.02 ± 0.12^bc^
LB102s	4.37 ± 0.11^abcd^	7.30 ± 0.16^fg^	5.82 ± 0.29^a^	7.69 ± 0.11^bc^
LBG0	6.39 ± 0.03^e^	nd	nd	nd
LBG100	4.51 ± 0.21^abcd^	6.78 ± 0.17^de^	7.75 ± 0.14^ cd^	nd
LBG101	4.19 ± 0.01^a^	7.75 ± 0.08^gh^	7.62 ± 0.17^cd^	7.52 ± 0.13^b^
LBG102	4.21 ± 0.21^ab^	7.80 ± 0.15^h^	7.88 ± 0.04^d^	7.47 ± 0.20^b^
LBG0s	6.26 ± 0.05^e^	nd	nd	nd
LBG100s	4.58 ± 0.07^bcd^	6.67 ± 0.16^d^	7.49 ± 0.01^cd^	nd
LBG101s	4.25 ± 0.07^abc^	5.59 ± 0.16^a^	6.74 ± 0.21^b^	6.83 ± 0.12^a^
LBG102s	4.14 ± 0.04^a^	7.66 ± 0.06^fgh^	7.25 ± 0.12^bc^	6.64 ± 0.09^a^
Statistics ANOVA. η^2^ [ −]				
GM	0.347	ns	ns	ns
C	0.989	0.969	0.978	0.992
S	ns	ns	0.358	ns

*nd* not detected, *ns* non-significant, *G* germination, *C* starter culture, *S* storage period.

All analyses were made in duplicate.

^a,b,c,d,e,f,g,h^Mean values in columns denoted by different letters differ significantly (*p* ≤ 0.05).

^1^Description as in Table 1.

At the time of consumption, probiotic products must contain an adequate number of viable cells, ranging from at least 10^6^ to 10^7^ CFU/mL, to exert their beneficial effects^21^. Here, by using the starter cultures YO-MIX 207 (BB/LB/BBG/LBG101) and ABY-3 (BB/ LB/BBG/LBG102), the recommended number of viable cells of the genera *Lactobacillus*, *Streptococcus,* and *Bifidobacterium* was achieved after fermentation of all tested samples (BBs/LBs and BBGs/LBGs) (Table 2). Most of these samples did not show a decrease in the bacterial population below 10^7^ CFU/mL during the storage period. The BB100, BBG100, and LB100 samples fermented with the Beaugel Soja 1 starter culture did not reach the recommended *Lactobacillus* cell count and showed values of 5.99, 5.86, and 5.73 log_10_ CFU/mL, respectively. All the tested samples of PBBs showed no significant effect of germination on the population of LAB and bifidobacteria. The 21-day refrigerated storage period also had no significant effect on the pH of all tested samples; however, it showed a slight effect (η^2^ ≈ 0.211–0.358) on changes in the viable population of bacteria.

In the present study, the required population level of LAB and bifidobacteria and the pH value recommended for fermented beverages were achieved for most of the tested samples. Moreover, these values were obtained by fermentation performed for 6 h. Most of the PBBs described in the literature exhibited a lower acidification rate, slow growth of probiotic bacteria, and prolonged fermentation time due to the low concentration of soluble carbohydrates^12,21^. A short fermentation time is more efficient and economically advantageous for use on a production scale.

G and non-G BBs and LBs fermented with the Beaugel Soja 1 starter culture showed a lower efficiency in obtaining the recommended pH and level of the bacterial population. This starter culture was the least diverse one with regard to microbial species and contained only *Lactobacillus* and *Streptococcus*. The other tested starter cultures (YO-MIX 207 and ABY-3) also contained the genus *Bifidobacterium*; this finding indicates that a greater diversity of microflora in the starter culture is conducive to a more effective fermentation of PBBs. This phenomenon may be due to the synergistic activities of microorganisms in complex starter cultures and the metabolism of carbohydrates in legumes. According to Adamberg et al.^22^, the strain balance and activities of microorganisms are determined by an interplay of different factors between consortium members, such as antagonism, competition for substrates, and symbiosis by cross-feeding. In their study, the authors showed that the growth of *L. paracasei* F8 was enhanced in the presence of *B. breve* 46. Similarly, in our study, a reduced concentration of *Lactobacillus* cells was observed only in samples fermented using the Beaugel Soja 1 starter culture, which did not contain bifidobacteria.

The samples fermented with Beaugel Soja 1 also had the slowest rate of pH decrease. According to Pokusaev et al.^23^, bifidobacteria metabolize carbohydrates more efficiently than LAB. The bifidobacterial pathway yields 2.5 mol of ATP, 1.5 mol of acetate, and 1 mol of lactate from 1 mol of fermented glucose. Homofermentative LAB produce 2 mol of ATP and 2 mol of lactic acid from 1 mol of glucose, while heterofermentative LAB produce 1 mol each of lactic acid, ethanol, and ATP from 1 mol of fermented glucose. Efficient milk fermentation can be achieved using the classic yogurt culture, which is characterized by protosymbiosis between *Streptococcus thermophilus* and *L. delbrueckii* subsp. *bulgaricus*^24^. The present study shows that for the fermentation of plant-based beverages, it is more effective to use starter cultures enriched with bifidobacteria, which can enhance fermentation rate and microbial proliferation.

### Carbohydrate content

Carbohydrates are the main component of legumes (55–65%) and consist mainly of starch, monosaccharides, disaccharides, and α-galactosides^25^. Humans and monogastric animals lack α-galactosidase required to hydrolyze α(1 → 6)glycosidic linkages; consequently, oligosaccharides remain undigested in their upper gastrointestinal tract. The undigested oligosaccharides are responsible for digestive discomfort^26^; therefore, it is desirable to remove these oligosaccharides during the production of legume-based products.

Table 3 shows the content of carbohydrates in the tested PBBs. BBs and BBGs showed the highest glucose content among all the carbohydrates tested. In BBs, germination was the primary factor that influenced the changes in the concentrations of glucose, sucrose, stachyose, and verbascose (η^2^ > 0.825). The germination process significantly reduced the content of glucose, sucrose, and verbascose and increased the content of stachyose. Fermentation with different starter cultures showed the greatest effect on reducing glucose and sucrose content in most of the tested BB and BBG samples; this decrease was directly caused by the metabolic activity of microorganisms present in the starter cultures used for fermentation.

**Table 3:** Carbohydrates content in the tested PBBs. Table shows mean values and standard deviations (SD) range, and statistics ANOVA (η^2^—coefficient indicating the extent of the effect of factors G, C and S).

Sample code^1^	Carbohydrates content [mg/kg]				
	Glucose	Sucrose	Raffinose	Stachyose	Verbascose
Bean-based beverages					
BB0	6.53 ± 0.10^f^	3.40 ± 0.24^f^	0.60 ± 0.11^a^	0.62 ± 0.04^a^	1.62 ± 0.03^bc^
BB100	4.73 ± 0.29^e^	2.67 ± 0.10^ef^	0.58 ± 0.12^a^	0.51 ± 0.03^a^	1.56 ± 0.09^bc^
BB101	4.33 ± 0.17^e^	2.11 ± 0.33^cde^	0.58 ± 0.11^a^	0.41 ± 0.01^a^	1.47 ± 0.05^bc^
BB102	4.29 ± 0.12^e^	2.16 ± 0.45^cde^	0.50 ± 0.03^a^	0.44 ± 0.01^a^	1.59 ± 0.11^bc^
BB0s	6.93 ± 0.28^f^	2.99 ± 0.03^ef^	0.59 ± 0.04^a^	0.65 ± 0.08^ab^	1.51 ± 0.02^bc^
BB100s	6.37 ± 0.53^f^	2.20 ± 0.12^cde^	0.74 ± 0.16^a^	0.64 ± 0.07^ab^	1.41 ± 0.01^b^
BB101s	5.97 ± 0.13^f^	2.16 ± 0.23^cde^	0.57 ± 0.05^a^	0.56 ± 0.08^a^	1.69 ± 0.12^c^
BB102s	4.87 ± 0.41^e^	1.59 ± 0.37^bcd^	0.55 ± 0.09^a^	0.40 ± 0.08^a^	1.55 ± 0.08^bc^
BBG0	2.58 ± 0.42^cd^	2.47 ± 0.17^de^	0.61 ± 0.02^a^	1.68 ± 0.29^f^	0.25 ± 0.08^a^
BBG100	2.25 ± 0.31^bcd^	0.91 ± 0.18^ab^	0.33 ± 0.06^a^	1.18 ± 0.09^cd^	0.27 ± 0.09^a^
BBG101	1.40 ± 0.02^ab^	1.45 ± 0.14^bc^	0.48 ± 0.09^a^	1.55 ± 0.13^cdef^	0.21 ± 0.01^a^
BBG102	2.21 ± 0.07^bcd^	1.02 ± 0.31^ab^	0.59 ± 0.22^a^	1.22 ± 0.02^cde^	0.22 ± 0.01^a^
BBG0s	3.03 ± 0.13^d^	2.46 ± 0.16^de^	0.62 ± 0.05^a^	1.67 ± 0.09^ef^	nd
BBG100s	2.94 ± 0.10^d^	0.79 ± 0.06^ab^	0.55 ± 0.12^a^	1.59 ± 0.03^def^	nd
BBG101s	1.09 ± 0.25^a^	0.79 ± 0.01^ab^	0.61 ± 0.04^a^	1.09 ± 0.05^bc^	0.19 ± 0.07^a^
BBG102s	1.89 ± 0.05^abc^	0.32 ± 0.11^a^	0.53 ± 0.14^a^	1.30 ± 0.22^cdef^	nd
Statistics ANOVA. η^2^ [ −]					
GM	0.918	0.825	ns	0.899	0.984
C	0.619	0.836	ns	0.394	ns
S	0.266	0.321	ns	ns	0.254
Lentil-based beverages					
LB0	12.49 ± 0.65^h^	1.20 ± 0.91^c^	0.25 ± 0.06^a^	1.15 ± 0.16^e^	2.65 ± 0.16^h^
LB100	11.53 ± 0.52^gh^	1.22 ± 0.01^c^	0.25 ± 0.04^a^	0.87 ± 0.10^abcd^	2.24 ± 0.14^fgh^
LB101	8.48 ± 0.49^f^	0.50 ± 0.05^b^	0.19 ± 0.01^a^	0.86 ± 0.19^abcd^	2.45 ± 0.15^gh^
LB102	4.68 ± 0.14^b^	0.40 ± 0.03^ab^	0.18 ± 0.01^a^	0.89 ± 0.12^abcd^	2.24 ± 0.08^fgh^
LB0s	10.29 ± 0.25^g^	1.08 ± 0.04^c^	0.23 ± 0.03^a^	1.09 ± 0.07^e^	1.78 ± 0.06^efg^
LB100s	6.33 ± 0.28^bcd^	0.33 ± 0.08^ab^	0.25 ± 0.02^a^	1.00 ± 0.03^e^	1.40 ± 0.05^cde^
LB101s	2.57 ± 0.64^a^	0.31 ± 0.01^ab^	0.14 ± 0.01^a^	0.99 ± 0.07^cd^	1.64 ± 0.07^ef^
LB102s	2.53 ± 0.57^a^	0.35 ± 0.07^ab^	0.12 ± 0.03^a^	0.97 ± 0.09^bcd^	1.60 ± 0.14^def^
LBG0	8.21 ± 0.24^ef^	1.21 ± 0.05^c^	0.91 ± 0.12^de^	1.19 ± 0.07^e^	0.90 ± 0.03^bcd^
LBG100	6.19 ± 0.08^bcd^	0.21 ± 0.08^a^	0.68 ± 0.06^bcd^	1.08 ± 0.03^e^	nd
LBG101	5.86 ± 0.16^bc^	0.52 ± 0.03^b^	0.71 ± 0.08^bcd^	0.98 ± 0.08^cd^	0.31 ± 0.01^ab^
LBG102	6.76 ± 0.33^cdef^	0.38 ± 0.08^ab^	0.73 ± 0.12^bcd^	0.61 ± 0.03^abc^	nd
LBG0s	7.79 ± 0.60^def^	1.07 ± 0.04^c^	0.99 ± 0.02^e^	1.01 ± 0.02^e^	0.78 ± 0.06^abc^
LBG100s	6.25 ± 0.31^bcd^	0.36 ± 0.08^ab^	0.61 ± 0.03^bc^	1.13 ± 0.09^e^	0.43 ± 0.06^ab^
LBG101s	6.49 ± 0.42^cde^	0.49 ± 0.02^b^	0.50 ± 0.05^b^	0.55 ± 0.03^a^	0.17 ± 0.06^a^
LBG102s	6.79 ± 0.62^cdef^	0.39 ± 0.03^ab^	0.78 ± 0.08^cde^	0.59 ± 0.08^ab^	nd
Statistics ANOVA. η^2^ [ −]					
GM	ns	ns	0.905	ns	0.901
C	0.487	0.714	0.440	0.468	0.389
S	0.219	ns	ns	ns	0.312

*nd* not detected, *ns* non-significant, *G* germination, *C* starter culture, *S* storage period.

All analyses were made in duplicate.

^a,b,c,d,e,f,g,h,i^Mean values in columns denoted by different letters differ significantly (*p* ≤ 0.05).

^1^Description as in Table 1.

LBs and LBGs also exhibited the highest glucose content among all the carbohydrates tested; however, compared to BB0 and BG0, the glucose content was almost twofold higher for LB0 (12.49 mg/kg) and more than threefold higher for LG0 (8.21 mg/kg), respectively (Table 3). Fermentation with different starter cultures was the main factor that affected the content of glucose (η^2^ = 0.487) and sucrose (η^2^ = 0.487) in LBs and led to a significant decrease in the content of these carbohydrates. In LBs, germination significantly reduced verbascose content and increased stachyose content, while fermentation significantly reduced the content of all the tested oligosaccharides. In all tested PBBs, the refrigerated storage period had the least effect on modifying the content of the analyzed carbohydrates.

According to Nkhata et al.^27^, the effect of germination on carbohydrates is largely dependent on the activation of hydrolytic and amylolytic enzymes; these enzymes decrease starch content and increase the content of simple sugars. Germination improves the digestibility of legumes by activating endogenous enzymes such as α-amylase. However, in the present study, germination led to a significant decrease in glucose content in the tested PBBs. The presence of glucose in the tested PBBs may result not only from the germination process used but also from other processing steps, including grinding. Ineffective grinding of the legumes may lead to a mass with a large particle size that will be retained on the sieves during the filtration process and will not be included in the beverage solution. This could reduce the concentration of dry matter in beverages and further result in a lower concentration of sugars^28,29^. Previous studies have shown that germinated BBs have a higher span and mean diameter d_4.3_ (span ≈ 2.24–2.35, d_4.3_ ≈ 76.8–84.2) than non-germinated BBs (span ≈ 1.90–2.00, d_4.3_ ≈ 38.2–47.0)^30^. This indicates the presence of larger particles or aggregates in the germinated beverage, which may directly result from the lower efficiency of the filtration process. The formation of protein aggregates is mainly observed in solutions prepared from legumes^31^. Germination leads to an increase in the availability of proteins^32^, which may result in more intensive formation of aggregates and, consequently, less effective grinding and filtration processes. Zahir et al.^33^ also observed the most intensive formation of protein aggregates in samples of germinated soybean preparations as compared to that in non-germinated ones.

### TPC and antioxidant capacity

Free radicals, reactive oxygen species, and reactive nitrogen species are generated by our body through various endogenous systems when exposed to different physiochemical or pathological conditions^34^. The use of various processing techniques, including germination and fermentation, may increase the TPC and antioxidant capacity of legumes following a reduction in the anti-nutritional factors (ANFs) and the production of new components with antioxidant properties^35,36^. We are also aware that the total phenolic content theoretically, but not necessarily, correlates with the antioxidant activity, although this is widely accepted as a screening parameter. The Folin-Ciocalteu method to quantify TPC has some limitations. This test is sensitive to pH, temperature, and reaction time as well as to inorganic and nonphenolic organic compounds (including reducing sugars) that react with the Folin reagent, thereby underestimating the phenol content^37^.

**Figure 1:**
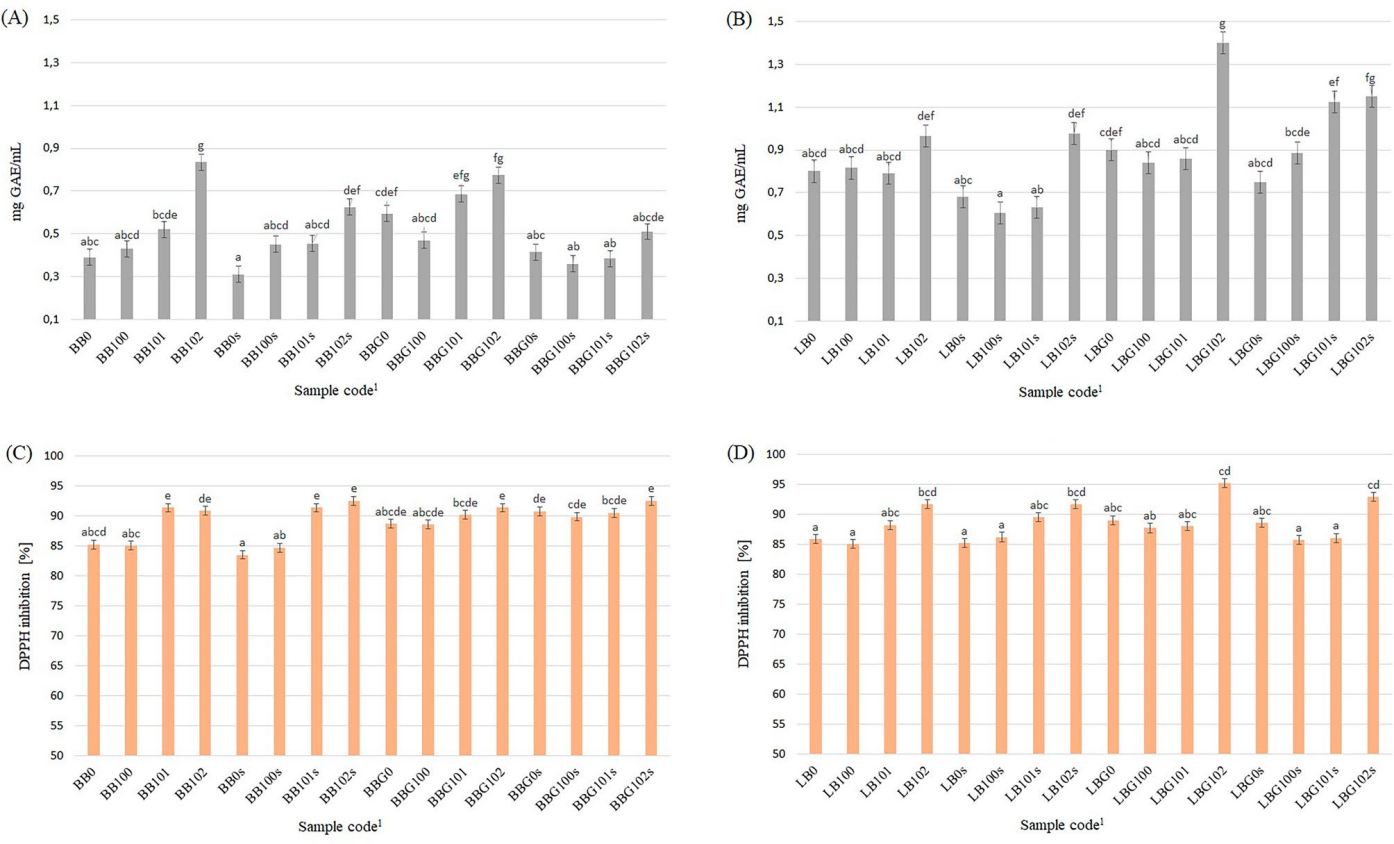
Total phenolic content of the tested BBs (**A**) and LBs (**B**) and DPPH radical scavenging activity of the tested BBs (**C**) and LBs (**D**). ^a,b,c,d,e,f,g^Mean values in columns denoted by different letters differ significantly (*p* ≤ 0.05). ^1^Description as in Table 1. All analyses were made in duplicate.

Figure 1 shows the TPC of the tested BBs (A) and LBs (B) and DPPH RSA of the tested BBs (C) and LBs (D). The results suggest that fermentation, choice of starter culture, germination, and storage conditions play crucial roles in determining the TPC of both BBs and LBs. These findings highlight the importance of these factors in modulating the antioxidant potential and potential health benefits of the tested products. The TPC of the base LB was twice as high as that of the base BB, which confirms the previous report that lentils naturally contain higher levels of phenolic compounds than beans^38,39^. Fermentation with the three different starter cultures was the primary factor that influenced the TPC of the tested PBBs (η^2^ = 0.652 for BBs and 0.651 for LBs). Fermentation with the starter culture ABY-3 (BB/BBG/LB/LBG102) was the most effective process. The present study showed that the refrigerated storage period significantly reduced the TPC in BBs, as indicated by the coefficient of determination (η2) value of 0.505. However, the refrigerated storage period did not affect the results obtained for LBs, thus implying that the phenolic content in LBs remained stable during refrigeration.

All the tested PBBs exhibited a relatively high DPPH scavenging capability (> 83%) (Fig. 1). The base BB and LB showed a similar ability to scavenge DPPH radicals (⁓85%). Fermentation with the three different starter cultures was the primary factor that influenced the DPPH RSA of the tested PBBs (η^2^ = 0.601 and 0.752 for BBs and LBs, respectively). Similar to the results obtained for TPC, fermentation with the starter culture ABY-3 (BB/BBG/LB/LBG102) was the most effective process. Germination significantly affected the obtained results only for the BBs (η^2^ = 0.285). Refrigerated storage had no effect on the DPPH RSA of all tested PBBs. This might be due to the presence of microorganisms and their metabolic activity, which also occurs during storage. LAB and bifidobacteria can produce bioactive peptides that increase the antioxidant capacity of the product^40,41^. Bioactive peptides are short amino acid sequences released from proteins by proteolysis, which are generated by enzymes produced by microorganisms^42^. Heydari et al.^43^ studied the effects of Iranian probiotic and commercial strains that included *B. lactis*, *L. acidophilus*, and *L. casei* on the functional properties of a water-soluble peptide extract. The tested microorganisms showed proteolytic activity during the 28-day cold storage period and significantly affected the formation of antioxidant, antimutagen and antibacterial peptides.

In the present study, the tested PBBs exhibited antioxidant properties derived from TPC and DPPH RSA. Compared to TPC, the DPPH RSA of the tested PBBs was more stable after the application of biotechnological processes and storage. This finding suggests that not only phenolic components but also other ingredients such as ascorbate, tocopherols, or carotenoids are responsible for the DPPH RSA of the studied PBBs^44^.

Fermentation was the primary factor that increased the TPC and DPPH RSA of the tested samples. Regardless of whether microbial fermentation is performed using fungi, yeasts, or bacteria, it affects the content of phenolic compounds in foods; moreover, metabolic activities are specific to the involved species or strains and depend on their array of enzymes. Among the fermented legumes, phenolic acid decarboxylase and esterase activities were reported in fermented cowpeas, lentils, and chickpeas^36^. In the present study, fermentation using the ABY-3 culture showed the greatest ability to increase the antioxidant properties of the tested beverages; this is because the culture contained the largest variety of microorganisms (*L. acidophilus* La-5, *B. animalis* subsp. *lactis* BB-12, *S. thermophilus*, and *L. delbrueckii* subsp. *bulgaricus*) among the tested starter cultures. Greater microbial diversity may present a broader enzyme array, which may increase the availability of antioxidant components, e.g., through the breakdown of ANFs^11^. In addition, the ABY-3 starter culture contains the strain *B. animalis subsp. lactis BB-12*, which is the world’s most documented probiotic *Bifidobacterium*^45^. These microorganisms produce a significant amount of bioactive peptides that may also exhibit antioxidant properties (e.g., iron-binding ferritin-like antioxidant protein)^46^. Previous studies also indicate that this strain has higher antioxidant activity than LAB^47,48^.

The effect of germination on the TPC and DPPH RSA of the tested PBBs varied depending on the analyzed raw material. Although these properties of BBGs and LBGs have not been previously investigated by other researchers, inconsistent results, depending on the tested raw material, were obtained for raw G and non-G legumes^49–51^. Therefore, the combination of germination and fermentation is the most beneficial approach to extend the effect on increasing the antioxidant properties of PBBs. This was also indicated by a previous study of Hubert et al.^52^, in which fermented soy germ extracts exhibited a higher inhibition effect against the superoxide anion radical, and lesser but significant DPPH radical scavenging effects compared to raw soy germ.

### Total share of FA and FA profile

**Figure 2:**
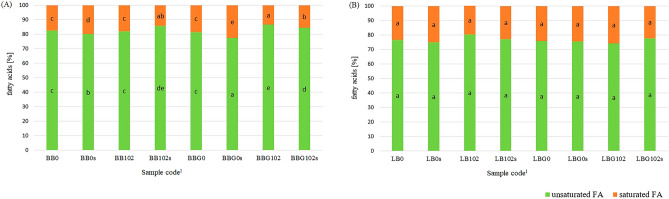
Total share of unsaturated and saturated fatty acids in the tested BBs (**A**) and LBs (**B**). ^a,b,c,d,e^Mean values in the group of FA denoted by different letters differ significantly (*p* ≤ 0.05). ^1^Description as in Table 1. All analyses were made in duplicate.

The content of FAs and the ratio between unsaturated fatty acids (UFAs) and saturated fatty acids (SFAs) are the important parameters to determine the nutritional value of fats^53,54^. Figure 2 and Supplementary Table S3 online shows the total share of UFAs and SFAs in the tested PBBs. UFAs dominated in all the tested PBBs, constituting 77.3–86.7% in BBs/BBGs and 74.1–80.3% in LBs/LBGs. Fermentation with the ABY-3 starter culture had a significant effect on the content of SFAs and UFAs in BBs/BBGs. Fermentation significantly increased the concentration of UFAs from 81.5% to 86.7% and simultaneously decreased the concentration of SFAs in BBG. In the remaining PBBs, fermentation, germination, and storage did not affect the total share of SFAs and UFAs.

In the FA profile of the tested PBBs, linolenic acid (C18:2 n-6c) was the dominant FA (Table 4). The other UFAs with a significant share in the tested PBBs were α-linolenic acid and oleic FAs. Only two SFAs, namely palmitic acid and stearic acid, had a significant share in the FA profile of the tested PBBs. The remaining FAs had a share of approximately 0.1% or less than that in the FA profile.

**Table 4:** Fatty acid profile (%) of the tested PBBs. Table shows mean values and standard deviations (SD) range, and statistics ANOVA (η2 – coefficient indicating the extent of the effect of factors G, C and S).

Sample code^1^	Palmitic FA C16:0	Stearic FA C18:0	Oleic FA C18:1 n9-c	Linoleic FA C18:2 n-6c	α-linolenic FA C18:3 n-3c
Bean-based beverages					
BB0	14.45 ± 0.35^c^	3.00 ± 0.00^c^	12.95 ± 0.21^b^	44.00 ± 0.14^d^	25.55 ± 0.35^b^
BB102	15.65 ± 0.35^ cd^	2.15 ± 0.07^a^	13.00 ± 0.00^b^	40.70 ± 0.14^a^	27.70 ± 0.14^c^
BB0s	16.90 ± 0.42^d^	2.95 ± 0.07^c^	12.10 ± 0.28^a^	43.15 ± 0.49^d^	24.90 ± 0.28^ab^
BB102s	11.30 ± 0.00^b^	2.50 ± 0.00^b^	13.70 ± 0.00^c^	41.85 ± 0.07^abc^	29.25 ± 0.07^d^
BBG0	15.15 ± 0.35^c^	3.25 ± 0.07^d^	12.80 ± 0.00^b^	43.75 ± 0.07^d^	25.05 ± 0.21^ab^
BBG102	9.60 ± 0.14^a^	2.55 ± 0.07^b^	12.75 ± 0.07^b^	42.95 ± 0.07^cd^	29.30 ± 0.14^d^
BBG0s	19.40 ± 0.57^d^	3.25 ± 0.07^d^	11.80 ± 0.00^a^	41.25 ± 0.64^ab^	24.30 ± 0.00^a^
BBG102s	11.85 ± 0.07^b^	2.50 ± 0.00^b^	12.90 ± 0.00^b^	41.95 ± 0.07^bc^	28.45 ± 0.07^c^
Statistics ANOVA. η2 [ −]84					
GM	ns	0.613	ns	ns	ns
C	0.550	0.929	0.452	0.306	0.914
S	ns	ns	ns	ns	ns
Lentil-based beverages					
LB0	17.50 ± 0.84^a^	5.75 ± 0.92^c^	26.15 ± 0.49^d^	39.90 ± 0.71^ab^	10.65 ± 1.06^ab^
LB102	15.70 ± 0.99^a^	2.15 ± 0.07^a^	24.70 ± 0.14^bcd^	42.35 ± 0.49^abc^	11.05 ± 0.07^b^
LB0s	18.40 ± 0.85^a^	6.65 ± 1.06^c^	25.35 ± 0.07^cd^	39.20 ± 1.13^a^	10.40 ± 0.85^ab^
LB102s	18.75 ± 0.92^a^	1.95 ± 0.07^a^	23.20 ± 0.28^bc^	41.30 ± 0.57^abc^	11.30 ± 0.14^b^
LBG0	19.20 ± 0.14^a^	4.75 ± 1.20^abc^	23.30 ± 0.71^bc^	43.85 ± 0.35^c^	8.80 ± 0.14^a^
LBG102	18.45 ± 0.78^a^	2.75 ± 0.07^ab^	19.65 ± 0.49^a^	42.35 ± 0.78^abc^	10.60 ± 0.14^ab^
LBG0s	19.45 ± 1.20^a^	5.10 ± 0.71^bc^	23.10 ± 0.00^b^	43.60 ± 0.42^bc^	8.75 ± 0.07^a^
LBG102s	16.55 ± 1.46^a^	2.20 ± 0.28^a^	19.35 ± 1.20^a^	45.10 ± 1.12^c^	11.35 ± 0.49^b^
Statistics ANOVA. η^2^ [ −]					
GM	ns	ns	0.872	0.626	0.426
C	ns	0.843	0.807	ns	0.613
S	ns	ns	ns	ns	ns

Other minor fatty acids in the fatty acid profile include 14:0, 15:0, 16:1, 17:0, 17:1, 20:0, 20:1, 20:3 n-3, 22:2, 24:0, 20:5 n-3.

*nd* not detected, *ns* non-significant, *G* germination, *C* starter culture, *S* storage period.

All analyses were made in duplicate.

^a,b,c,d^Mean values in columns denoted by different letters differ significantly (*p* ≤ 0.05).

^1^Description as in Table 1.

Fermentation with the ABY-3 starter culture was the primary factor that affected the FA profile of BBs and BBGs (η^2^ ≈ 0.306–0.929) (Table 4). Fermentation significantly modified the profile of n-3 and n-6 FAs by increasing the share of α-linolenic acid n-3 and reducing the share of linoleic acid n-6. Moreover, fermentation significantly reduced the content of palmitic acid in BBGs and reduced stearic acid content in BBs and BBGs. Germination significantly increased the share of only stearic acid in BBG (from 3.0 to 3.25%).

Both fermentation and germination showed a significant effect on the FA profile of the tested LBs and LBGs. Fermentation with the ABY-3 starter culture significantly reduced the share of stearic acid and oleic acid and increased the share of α-linolenic acid (Table 4). Germination significantly reduced the share of oleic acid and α-linolenic acid and increased the share of linoleic acid. The refrigerated storage period had no effect on the FA profile of all the tested PBBs.

UFAs dominated the FA profile of the tested PBBs, thus making beans and lentils suitable for nutritional applications. A similar trend of the dominance of UFAs in FA profiles was observed for nonfermented legume-based beverages, including BB^18,55^ and soy-based beverages^56^. In the present study, among the SFAs, only palmitic acid and stearic acid had a significant share, with palmitic acid being the predominant SFA; palmitic acid was also identified in raw beans and lentil seeds^18,57^. The FA profile in the fats of the legume seeds, however, differs considerably between the legume species and even between their varieties^58^. This is reflected directly by the FA profile of PBBs prepared from them.

During the production of legume-based beverages, it is beneficial to use processes that will contribute to increase the share of n-3 UFAs and/or reduce the share of SFAs in the FA profile. In the tested PBBs, the use of fermentation to modify the FA profile was more efficient than the use of germination. Fermentation using the ABY-3 starter culture significantly increased the share of α-linolenic acid and decreased the share of stearic acid in the FA profile of BBs/BBGs and LBs/LBGs. In all the tested PBBs, a multidirectional modification of the content of the remaining analyzed FAs was also observed. According to Adebo et al.^59^, the observed increase and decrease in these fat-related constituents after fermentation suggest selective lipase activities. While these lipolytic enzymes could have contributed to lipid dissociation and increased the extractability of fat-related constituents, the same enzymes could have also exerted selective reductive activities, perhaps using these fat-related components as carbon sources.

In the tested PBBs, germination mainly influenced the modification of the share of UFAs in the FA profile of LBs and LBGs by reducing the share of oleic and α-linolenic acids. The decrease in FAs could be due to hydrolysis during seed germination, where they were used to produce the necessary energy for biochemical and physicochemical modifications^60^. A significant modification of the UFAs in the FA profile during germination was also reported in raw lentil seeds^60,61^.

### Positional distribution of FAs in TAGs

The intramolecular structure of TAGs in terms of the position of the FA chain in the glycerol backbone (sn1, sn2, and sn3 positions) influences the digestion and absorption of FAs^62^. Pancreatic lipase, which is responsible for the hydrolysis of TAGs, hydrolyzes the sn1 and sn3 positions of dietary TAGs, thereby producing free FAs and 2-monoglyceride. Structural rearrangement of 2-monoglycerides can occur during digestion, resulting in complete degradation into glycerol and free FAs^63^. In plants, TAGs are synthesized with structural specificity: SFAs can be found mostly in terminal positions, while UFAs are located at the sn-2 position^64^. There are very few scientific reports on the effect of biotechnological processes on the distribution of FAs in TAGs in food, and these reports are related to the use of lipids in human milk fat substitutes^65^.

Tables 5 and 6 show the positional distribution of sn2 FAs in TAGs and the positional distribution of sn1,3 FAs in TAGs in the tested PBBs, respectively. In all nonfermented PBBs, the highest share of palmitic acid was found at the sn2 position in TAGs. Among UFAs, oleic acid had the largest share of all sn2 FAs. Similarly, in the sn1,3 position in TAGs, PUFAs, i.e., linoleic acid and α-linolenic acid, had the highest share. Fermentation was the main factor that influenced the changes in the distribution of sn2 and sn1,3 FAs in TAGs. The application of fermentation significantly reduced the share of SFAs and monounsaturated fatty acids (MUFAs), increased the share of PUFAs in the sn2 position in TAGs, increased SFAs and MUFAs, and decreased PUFAs in the sn1,3 position in TAGs for BB/BBG/LB102. The exception was LBGs, where the share of PUFAs in the sn2 position in TAGs decreased for linoleic and α-linolenic acids, and consequently, the share of α-linolenic acid in the sn1,3 position in TAGs increased. Moreover, after the fermentation, previously absent stearic acid in BBs and BBGs and myristic acid in LBs and LBGs appeared among the sn2 and sn1,3 FAs in TAGs. The refrigerated storage period had no effect on FA distribution in TAGs in most of the tested PBBs.

**Table 5:** Positional distribution of sn2 FA in TAGs (%) in the tested PBBs. Table shows mean values and standard deviations (SD) range, and statistics ANOVA (η^2^—coefficient indicating the extent of the effect of factors G, C and S).

Sample code^1^	Myristic FA C14:0	Palmitic FA C16:0	Stearic FA C18:0	Oleic FA C18:1 n9-c	Linoleic FA C18:2 n-6c	α-linolenic FA C18:3 n-3c
Bean-based beverages						
BB0	nd	52.66 ± 1.29^d^	nd	39.42 ± 1.10^c^	4.62 ± 0.47^a^	3.30 ± 0.16^a^
BB102	nd	5.72 ± 0.64^a^	10.84 ± 0.64^a^	26.71 ± 1.00^b^	32.20 ± 0.14^d^	24.53 ± 1.09^d^
BB0s	nd	52.25 ± 1.39^d^	nd	40.33 ± 0.49^c^	4.40 ± 0.16^a^	3.02 ± 0.18^a^
BB102s	nd	20.56 ± 1.46^c^	39.19 ± 1.36^c^	21.21 ± 1.09^a^	11.59 ± 0.47^b^	7.45 ± 0.55^b^
BBG0	nd	51.20 ± 1.58^d^	nd	40.63 ± 1.60^c^	4.74 ± 0.17^a^	3.43 ± 0.21^a^
BBG102	nd	14.55 ± 0.61^b^	17.43 ± 0.72^b^	24.72 ± 1.20^ab^	25.50 ± 0.99^c^	17.80 ± 0.83^c^
BBG0s	nd	52.55 ± 0.92^d^	Nd	39.99 ± 1.05^c^	4.42 ± 0.15^a^	3.05 ± 0.09^a^
BBG102s	nd	6.71 ± 0.35^a^	8.58 ± 0.86^a^	27.97 ± 1.27^b^	33.15 ± 1.34^d^	23.59 ± 0.91^d^
Statistics ANOVA. η^2^ [ −]84						
GM	–	ns	ns	ns	ns	ns
C	–	0.958	0.592	0.941	0.782	0.745
S	–	ns	ns	ns	ns	ns
Lentil-based beverages						
LB0	nd	34.10 ± 1.43^c^	13.60 ± 0.62^c^	30.80 ± 1.30^b^	16.20 ± 0.83^a^	5.30 ± 0.23^b^
LB102	3.70 ± 0.28^a^	12.80 ± 0.99^a^	4.40 ± 0.28^a^	27.70 ± 1.53^ab^	42.10 ± 1.39^d^	9.30 ± 0.31^d^
LB0s	nd	34.44 ± 1.16^c^	14.19 ± 0.69^c^	30.53 ± 1.45^ab^	15.93 ± 0.25^a^	4.93 ± 0.22^b^
LB102s	6.50 ± 0.42^c^	19.60 ± 1.13^b^	6.20 ± 0.47^a^	26.80 ± 1.06^ab^	33.30 ± 1.13^c^	7.60 ± 0.18^c^
LBG0	nd	31.60 ± 1.98^c^	12.90 ± 0.82^c^	29.80 ± 0.98^ab^	20.60 ± 0.78^b^	5.10 ± 0.23^b^
LBG102	10.90 ± 0.35^d^	33.10 ± 1.84^c^	10.10 ± 0.37^b^	25.50 ± 1.40^a^	16.80 ± 0.64^ab^	3.60 ± 0.13^a^
LBG0s	nd	33.63 ± 0.98^c^	12.69 ± 0.52^c^	29.33 ± 1.10^ab^	19.47 ± 0.96^ab^	4.88 ± 0.18^b^
LBG102s	5.40 ± 0.42^b^	17.50 ± 1.27^ab^	4.60 ± 0.20^a^	25.40 ± 1.43^a^	39.40 ± 1.24^d^	7.70 ± 0.66^c^
Statistics ANOVA. η^2^ [ −]						
GM	ns	ns	ns	0.370	ns	ns
C	0.792	0.608	0.819	0.799	0.543	0.372
S	ns	ns	ns	ns	ns	ns

*nd* not detected, *ns* non-significant, *G* germination, *C* starter culture, *S* storage period.

All analyses were made in duplicate.

^a,b,c,d^Mean values in columns denoted by different letters differ significantly (*p* ≤ 0.05).

^1^Description as in Table 1.

**Table 6:** Positional distribution of sn1,3 FA in TAGs (%) in the tested PBBs. Table shows mean values and standard deviations (SD) range, and statistics ANOVA (η^2^—coefficient indicating the extent of the effect of factors G, C and S).

Sample code^1^	Myristic FA C14:0	Palmitic FA C16:0	Stearic FA C18:0	Oleic FA C18:1 n9-c	Linoleic FA C18:2 n-6c	α-linolenic FA C18:3 n-3c
Bean-based beverages						
BB0	nd	2.93 ± 0.20^a^	nd	6.52 ± 0.55^a^	56.80 ± 1.40^d^	33.75 ± 0.96^bc^
BB102	nd	21.94 ± 0.85^e^	2.83 ± 0.18^a^	12.68 ± 0.99^c^	34.31 ± 1.10^a^	28.24 ± 0.91^a^
BB0s	nd	2.75 ± 0.20^a^	nd	6.00 ± 0.18^a^	56.59 ± 1.58^d^	34.66 ± 0.92^c^
BB102s	nd	8.35 ± 0.59^b^	nd	9.79 ± 0.72^b^	45.40 ± 0.98^c^	36.41 ± 0.82^c^
BBG0	nd	3.92 ± 0.48^a^	nd	6.32 ± 0.47^a^	56.61 ± 1.44^d^	33.15 ± 1.00^c^
BBG102	nd	11.60 ± 0.66^c^	2.90 ± 0.16^a^	12.20 ± 0.93^bc^	40.26 ± 1.16^b^	33.04 ± 0.95^bc^
BBG0s	nd	3.68 ± 0.18^a^	nd	5.70 ± 0.52^a^	56.30 ± 1.54^d^	34.32 ± 0.81^bc^
BBG102s	nd	16.79 ± 0.92^d^	3.45 ± 0.24^b^	12.64 ± 0.69^c^	36.67 ± 0.82^ab^	30.45 ± 0.61^ab^
Statistics ANOVA. η^2^ [ −]84						
GM	–	ns	0.613	ns	ns	ns
C	–	0.722	0.862	0.917	0.898	ns
S	–	ns	ns	ns	ns	ns
Lentil-based beverages						
LB0	nd	9.20 ± 0.27^a^	1.90 ± 0.13^c^	23.80 ± 1.05^d^	51.80 ± 1.57^cd^	13.30 ± 0.58^bcd^
LB102	3.90 ± 0.17^c^	12.80 ± 0.79^b^	3.20 ± 0.14^d^	28.50 ± 1.27^e^	42.30 ± 1.19^a^	9.30 ± 0.54^a^
LB0s	nd	9.02 ± 0.33^a^	1.60 ± 0.56^bc^	23.18 ± 0.92^cd^	52.56 ± 0.80^cd^	13.65 ± 0.83^cd^
LB102s	1.90 ± 0.07^a^	18.30 ± 0.91^c^	0.20 ± 0.00^a^	21.40 ± 0.89^bcd^	45.00 ± 1.00^ab^	13.20 ± 0.61^bcd^
LBG0	nd	13.00 ± 0.69^b^	0.70 ± 0.06^ab^	20.10 ± 0.74^bc^	55.50 ± 1.68^d^	10.70 ± 0.51^ab^
LBG102	2.50 ± 0.23^b^	11.10 ± 0.85^b^	0.90 ± 0.10^ab^	16.20 ± 0.47^a^	54.10 ± 1.40^cd^	15.20 ± 0.58^d^
LBG0s	nd	12.52 ± 0.59^ab^	0.53 ± 0.08^a^	19.37 ± 0.54^ab^	56.45 ± 1.60^d^	11.14 ± 0.83^abc^
LBG102s	2.30 ± 0.17^ab^	16.10 ± 0.82^c^	1.00 ± 0.30^abc^	16.60 ± 0.38^a^	49.80 ± 1.17^bc^	14.20 ± 0.99^d^
Statistics ANOVA. η^2^ [ −]						
GM	ns	ns	0.340	0.703	0.703	ns
C	0.898	0.443	ns	ns	0.718	ns
S	ns	ns	0.293	ns	ns	ns

*ns* non-significant, *G* germination, *C* starter culture, *S* storage period.

All analyses were made in duplicate.

^a,b,c,d,e^Mean values in columns denoted by different letters differ significantly (*p* ≤ 0.05).

^1^Description as in Table 1.

To date, it remains unclear how fermentation using complex starter cultures affects the changes in the distribution of FAs in the TAGs of PBBs. Ziarno et al.^18^ analyzed the effect of fermentation of BBs on the positional distribution of FAs in TAGs by using monocultures of *Lactobacillus*. After fermentation, the BBs usually showed a lower share of palmitic and stearic acid in the sn2 position. The fermentation also increased the share of oleic acid in the sn2 position as compared to that in BBs obtained from germinated beans. Fermented beverages showed an average higher share of PUFAs (linoleic and α-linolenic acids) than that observed in the nonfermented PBBs. The present study showed similar results for SFAs and PUFAs.

The obtained results indicate that the fermentation of legume-based beverages increases the share of PUFAs in the sn2 position in TAGs and simultaneously increases the share of SFAs and MUFAs in the sn1,3 position in TAGs. This is beneficial from a nutritional point of view, as SFAs and MUFAs will be first hydrolyzed by pancreatic lipase and separated from TAGs. These FAs will be less efficiently absorbed in the intestine because they can react with free calcium ions to form insoluble calcium salts, which are then excreted in the feces^66^. PUFAs located mainly at the sn2 position in TAGs will be mostly absorbed as monoacylglycerols. PUFAs are referred to as dietary essential FAs because humans cannot synthesize these molecules; therefore, they must be supplied with the diet^67^.

## Conclusions

The development of legume-based beverages is essential to provide consumers with PBBs with a high nutritional value, particularly those with a protein content similar to milk. Although this segment of PBBs is still poorly researched, previous studies indicate limitations related to their consumption, such as the presence of ANFs or barriers related to taste and texture. The present study demonstrated that the use of natural biotechnological processes in the production of newly developed beverages made from beans and lentils enables to increase their nutritional properties. The use of germination and fermentation improved the antioxidant properties, modified the oligosaccharide content, and altered the profile and positional distribution of FAs in TAGs of the tested PBBs. Importantly, the tested beverages were a good matrix for the fermentation process using LAB and bifidobacteria, including the probiotic species.

Based on the findings of the present study, we recommend that the designing of innovative legume-based beverages should include the use of fermentation and germination processes that will allow to obtain products that serve as carriers of probiotic bacteria and have improved nutritional properties. Moreover, it is recommended to use multispecies starter cultures containing both LAB and bifidobacteria in the fermentation of legume-based beverages, which enables a more efficient process. These products can directly meet the market demand for high-quality plant products as a substitute for animal products. Future research should focus on analyzing the properties of PBBs derived from other legumes and conducting a broad assessment of their sensory properties, which may be the most important factor limiting the consumption of PBBs by consumers. Moreover, the presented study considered selected health-promoting and functional properties of BBs and LBs as plant products with high nutritional value. These beverages are poorly described in the literature, so we recommend that future research should focus on the analysis of their other properties, in particular the quantity and quality of proteins and amino acids. This will allow for an in-depth analysis of these products also in terms of their suitability as analogues of cow's milk.

### Supplementary Information

Supplementary Tables.

## Data Availability

The datasets generated during and/or analysed during the current study are available from the corresponding author on reasonable request.
