# Supplementary material for: Use of natural biotechnological processes to modify the nutritional properties of bean-based and lentil-based beverages
Source: Sci Rep. 2023 Oct 9;13:16976. doi: 10.1038/s41598-023-44239-8 (PMC10562390; doi:10.1038/s41598-023-44239-8)
Supplement: Supplementary file 1 — Supplementary Tables. [file 41598_2023_44239_MOESM1_ESM.pdf]

USE OF NATURAL BIOTECHNOLOGICAL PROCESSES TO MODIFY THE NUTRITIONAL PROPERTIES OF LEGUME-BASED BEVERAGES

Patrycja Cichońska[1*], Joanna Bryś[2], Małgorzata Ziarno[1]

Table S1. Nutritional values of the tested beans and lentils.

Nutritional values per 100 g		White kidney beans	Brown lentils
Energy	[kcal]	295	353
	[kJ]	1237	1477
Total Fat [g]		2.3	1.1
- Saturated fat [g]		0.5	0.2
Total carbohydrates [g]		37.0	28.6
- Sugars [g]		9.7	2.0
Protein		19.0	25.8
Salt		<0.05	<0.05

Table S2. Physicochemical characteristics of the tested PBBs.

Sample code[1]	Dry Mater [g/100 g]	Protein [g/100 g]	Total Fat [g/100 g]
Bean-based beverages			
BB0	7.63	2.24	0.60
BBG0	6.82	2.59	0.60
Lentil-based beverages			
LB0	7.21	2.09	0.15
LBG0	5.69	2.37	0.15

[1] Description as in Table 1.

Table S3. Total share of unsaturated and saturated fatty acids in the tested PBB. Table shows mean values and standard deviations (SD) range, and statistics ANOVA (η2 – coefficient indicating the extent of the effect of factors G, C and S).

Sample code[1]	unsaturated FA [%]	saturated FA [%]
Bean-based beverages		
BB0	82.50 ± 0.28 [c]	17.50 ± 0.28 [c]
BB102	82.05 ± 0.21 [c]	17.95 ± 0.21 [c]
BB0s	80.15 ± 0.49 [b]	19.85 ± 0.49 [b]
BB102s	85.75 ± 0.07 [de]	14.25 ± 0.07 [de]
BBG0	81.60 ± 0.28 [c]	18.40 ± 0.28 [c]
BBG102	86,75 ± 0.21 [e]	13.25 ± 0.21 [e]
BBG0s	77.35 ± 0.64 [a]	22.65 ± 0.64 [a]
BBG102s	84.50 ± 0.00 [d]	15.50 ± 0.00 [d]
Statistics ANOVA. η2 [-]		
G	ns	ns
C	0.606	0.606
S	ns	ns
Lentil-based beverages		
LB0	76.70 ± 0.85 [a]	23.30 ± 0.85 [a]
LB102	80.30 ± 0.85 [a]	19.70 ± 0.85 [a]
LB0s	74.95 ± 0.21 [a]	25.05 ± 0.21 [a]
LB102s	77.30 ± 0.85 [a]	22.70 ± 0.85 [a]
LBG0	75.95 ± 1.06 [a]	24.05 ± 1.06 [a]
LBG102	74.10 ± 1.41 [a]	25.90 ± 1.41 [a]
LBG0s	75.45 ± 0.49 [a]	24.55 ± 0.49 [a]
LBG102s	77.80 ± 1.53 [a]	22.20 ± 1.53 [a]
Statistics ANOVA. η2 [-]		
G	ns	ns
C	ns	ns
S	ns	ns

[a, b, c, d, e] - mean values in columns denoted by different letters differ significantly ($p \leq 0.05$).

[1] Description as in Table 1.

Explanations: ns – non-significant; G – germination; C – starter culture; S – storage period

All analyses were made in duplicate.
